# Epidemiology and population structure of *Haemophilus influenzae* causing invasive disease

**DOI:** 10.1099/mgen.0.000723

**Published:** 2021-12-13

**Authors:** Anna Carrera-Salinas, Aida González-Díaz, Laura Calatayud, Julieta Mercado-Maza, Carmen Puig, Dàmaris Berbel, Jordi Càmara, Fe Tubau, Imma Grau, M. Ángeles Domínguez, Carmen Ardanuy, Sara Martí

**Affiliations:** ^1^​ Microbiology Department, Bellvitge University Hospital, IDIBELL-UB, Barcelona, Spain; ^2^​ Research Network for Respiratory Diseases (CIBERES), ISCIII, Madrid, Spain; ^3^​ Infectious Diseases Department, Bellvitge University Hospital, IDIBELL-UB, Barcelona, Spain; ^4^​ Spanish Network for Research in Infectious Diseases (REIPI), ISCIII, Madrid, Spain; ^5^​ Department of Pathology and Experimental Therapeutics, School of Medicine, University of Barcelona, Barcelona, Spain; ^6^​ Department of Medicine, School of Medicine, University of Barcelona, Barcelona, Spain

**Keywords:** epidemiology, *Haemophilus influenzae*, invasive disease, structure population

## Abstract

This study provides an update on invasive *Haemophilus influenzae* disease in Bellvitge University Hospital (2014–2019), reporting its evolution from a previous period (2008–2013) and analysing the non-typeable *H. influenzae* (NTHi) population structure using a clade-related classification. Clinical data, antimicrobial susceptibility and serotyping were studied and compared with those of the previous period. Population structure was assessed by multilocus sequence typing (MLST), SNP-based phylogenetic analysis and clade-related classification. The incidence of invasive *H. influenzae* disease remained constant between the two periods (average 2.07 cases per 100 000 population), while the 30 day mortality rate decreased (20.7–14.7 %, respectively). Immunosuppressive therapy (40 %) and malignancy (36 %) were the most frequent comorbidities. Ampicillin and fluoroquinolone resistance rates had increased between the two periods (10–17.6 % and 0–4.4 %, respectively). NTHi was the main cause of invasive disease in both periods (84.3 and 85.3 %), followed by serotype f (12.9 and 8.8 %). NTHi displayed high genetic diversity. However, two clusters of 13 (*n*=20) and 5 sequence types (STs) (*n*=10) associated with clade V included NTHi strains of the most prevalent STs (ST3 and ST103), many of which showed increased frequency over time. Moreover, ST103 and ST160 from clade V were associated with β-lactam resistance. Invasive *H. influenzae* disease is uncommon, but can be severe, especially in the elderly with comorbidities. NTHi remains the main cause of invasive disease, with ST103 and ST160 (clade V) responsible for increasing β-lactam resistance over time.

## Data Summary

Sequence reads were deposited in the European Nucleotide Archive (ENA) under the project accession number PRJEB42971 (Dataset S1, available in the online version of the article).

**Impact Statement**

*
Haemophilus influenzae* is an opportunistic pathogen in the human respiratory tract that may cause community-acquired invasive infections with high mortality rates. Moreover, the rise in β-lactam resistance due to β-lactamase production is cause for concern. We provide an update on the current epidemiology of invasive *H. influenzae* disease, as well as an analysis of the heterogeneous non-typable *H. influenzae* (NTHi) population structure using a clade-related classification based on accessory genome analysis. Multilocus sequence typing (MLST) is useful for clustering capsulated *H. influenzae* isolates because they are clonal and have few sequence types (STs), but it is not the best method for studying the heterogeneous NTHi population. The use of a clade-related classification based on the presence or absence of some surface-associated proteins and virulence determinants is a valuable tool to address this issue. Interestingly, we have observed that despite the genetic diversity, more than half of the invasive NTHi isolates shared a common phylogenetic origin, clustering them in the same clade. Moreover, these isolates showed an increased frequency over time and were linked to β-lactamase production. This should raise our awareness of the possibility of clonal dissemination, and we should consider monitoring the evolution of these clones in invasive disease.

## Introduction

*Haemophilus influenzae* is a Gram-negative coccobacillus that colonizes the human nasopharynx and throat in up to 80 % of children and 20–30 % of adults [1, 2]. Capsulated strains produce different capsular polysaccharides (identified as serotypes a to f), a distinctive feature that distinguishes them from the non-capsulated strains that are commonly known as non-typeable *H. influenzae* (NTHi) [3].

Despite the usual asymptomatic interaction with humans, *H. influenzae* is responsible for respiratory infections ranging from otitis media, sinusitis and conjunctivitis to deeper infections such as pneumonia and exacerbations of chronic obstructive pulmonary disease (COPD) and even invasive disease [4–7]. Before the widespread introduction of the *H. influenzae* type b conjugate vaccine, this serotype was the most common cause of invasive disease. Vaccine implementation has produced an epidemiological shift, with most invasive infections currently occurring in elderly patients with underlying conditions and caused mainly by NTHi followed by non-type-b serotypes [3, 4, 8]. As the epidemiology of invasive *H. influenzae* disease evolves, concerns are now focused on non-vaccine-preventable isolates and the growing proportion of β-lactam resistance by the acquisition of β-lactamases and alterations in penicillin-binding proteins (PBPs) [9, 10].

The structure of the bacterial population provides information on the evolution of a bacterial species, its genetic diversity and the dynamics of antimicrobial resistance determinants and pathogenic mechanisms [11, 12]. Classification based on multilocus sequence typing (MLST) is useful for clustering capsulated *H. influenzae* isolates, as they are highly clonal and associated with few sequence types (STs). By contrast, NTHi strains show high genetic heterogeneity [13]. The classification of NTHi into clades according to the presence or absence of some surface-associated proteins and virulence determinants, as previously proposed [12, 14], could be a valuable tool to address this issue.

Epidemiological surveillance of invasive *H. influenzae* clones, along with their antibiotic resistance profile and genetic variability, is essential to design useful prevention strategies. In this study, we provide an update on the epidemiology of invasive *H. influenzae* disease in Bellvitge University Hospital (2014–2019), monitoring changes from a previous period (2008–2013) [15].

## Methods

### Study design and bacterial strains

A laboratory-based surveillance study on the epidemiology of invasive *H. influenzae* disease between 2008 and 2019 (first period: 2008–2013 [15]; second period: 2014–2019) was undertaken. This study was conducted at Bellvitge University Hospital, a tertiary care centre for adult patients that provides health assistance to the southern area of Barcelona, Spain. Cases of invasive disease were defined as the isolation of *H. influenzae* from the blood, cerebrospinal fluid or pleural fluid (Table S1), the same sample types considered in the previous study period (2008–2013) [15] to facilitate comparison of the results. All clinical *H. influenzae* isolates were identified by mass spectrometry using MALDI-TOF (MALDI Biotyper, Bruker).

The medical records of patients with invasive *H. influenzae* disease were reviewed to obtain demographic and clinical data, including information on gender, age, source of infection, underlying conditions and 30 day mortality. The incidence of invasive *H. influenzae* disease was expressed as the number of cases per 100 000 population and was estimated using the adult population that received health coverage in Bellvitge University Hospital as the denominator, according to the Statistical Institute of Catalonia (https://www.idescat.cat).

### Antimicrobial susceptibility testing

Antimicrobial susceptibility was assessed by the microdilution method, using commercial panels (STRHAE2, Sensititre) and following the recommendations for clinical breakpoints of the Clinical and Laboratory Standards Institute (CLSI) [16]. β-lactamase activity was measured using the chromogenic cephalosporin method (Cefinase disc, BD).

### Whole genome sequencing

All 68 invasive *H. influenzae* isolates identified during the second period (2014–2019) were subjected to whole-genome sequencing (WGS) for serotyping, MLST classification and the study of resistance-related mutations. Genomic DNA was extracted using the QIAamp DNA Mini Kit (Qiagen) and quantified by the QuantiFluor dsDNA System (Promega). Nextera XT was used to prepare the libraries, followed by paired-end sequencing on a MiSeq platform (Illumina). Sequences were assembled with the INNUca v4.2 pipeline (github.com/BUMMI/INNUca) using default parameters. MLST was performed using the mlst v2.4 software (Seemann T, mlst Github, github.com/tseemann/mlst). New allele and ST numbers were registered in PubMLST (pubmlst.org). Full MST algorithm with PHYLOViZ [17] was used to analyse the relationship between the STs. *In silico* serotyping was conducted using hicap (github.com/scwatts/hicap) [18]. The mechanisms of acquired resistance to antibiotics were screened using ResFinder [19]. The screening of mutations in genes involved in antibiotic resistance was performed using Geneious R9 (Biomatters), with the closed genome of *H. influenzae* Rd KW20 (NC_000907) used as the reference. For phylogenetic analysis, core-SNPs were extracted with Snippy’s core module (snippy-core). Subsequently, a novel core-SNP phylogenetic tree, using RAxML-NG [20], was built using a discrete GAMMA model of rate heterogeneity, and 100 bootstrap replicates to determine the phylogeny of the 68 strains included in this study and the strains of clade V, separately. Relative bootstrap values below 0.75 indicated poor support [21]. The Hi375 strain (CP009610) was used as a reference. The phylogenetic trees were visualized using Microreact (microreact.org). The core-genome SNP alignment obtained with Snippy’s core module was subjected to the prediction of recombinant regions using the Gubbins v2.3.1 software to assess the proportion of sites affected by recombination, which was expressed as the relative impact of recombination and mutation (r/m) [22].

### NTHi clade analysis

NTHi isolates were grouped into clades, as previously proposed [12, 14]. A total of 213 NTHi genomes were included in this analysis: 98 genomes from De Chiara *et al.* [12], 57 genomes from Pinto *et al.* [14], and 58 genomes from this study. Six NTHi clades (I to VI) were defined using patho_typing (github.com/B-UMMI/patho_typing) based on the presence or absence of 17 gene sequences [14]. Phylogenetic analysis was performed by constructing an assembly-based core-SNP phylogenetic tree, using Parsnp from the Harvest suite [23] with default parameters, apart from parameter -C, which was adjusted to 5000 to maximize the reference coverage. The Hi375 strain (CP009610) was used as a reference. Tree visualization was performed using the ggtree R package [24] (available at github.com/micro-bellvitge/Phylogenetic-analysis-invasive-HINF).

### Statistical analysis

Statistical analyses were carried out using the GraphPad Prism 5 software, applying Fisher’s exact test or unpaired *t*-test when appropriate. *P*-values<0.05 were considered statistically significant.

## Results

### Clinical characteristics

A total of 8191* H. influenzae* strains were isolated from adult patients admitted to Bellvitge University Hospital (2008–2019). Of these, 150 (1.8 %) were responsible for invasive disease, most of which had been isolated from blood (88.0 %) and less frequently from cerebrospinal and pleural fluids (8.7 and 3.3 %, respectively). In the first period (2008–2013), 82 of the 3433 (2.4 %) *H. influenzae* isolates caused invasive disease, compared to 68 of the 4758 isolates (1.4 %) in the second period (2014–2019).

The overall incidence of invasive *H. influenzae* disease in the adult population was 2.07 cases per 100 000 population, with no significant differences between the two periods (2.12 and 2.02 episodes per 100 000 population, respectively). By age groups, the overall incidence was lower in young adults (≤64 years) than among those aged 65 or older (0.89 and 6.90 cases per 100 000, respectively) (*P*-value<0.0001), as observed in each study period separately (1.10 and 6.80 cases per 100 000 in the first period, and 0.68 and 6.97 per 100 000 in the second period). The incidence in young adults decreased in the second period (*P*-value=0.03), while the incidence in older adults remained stable.

**Fig. 1.**
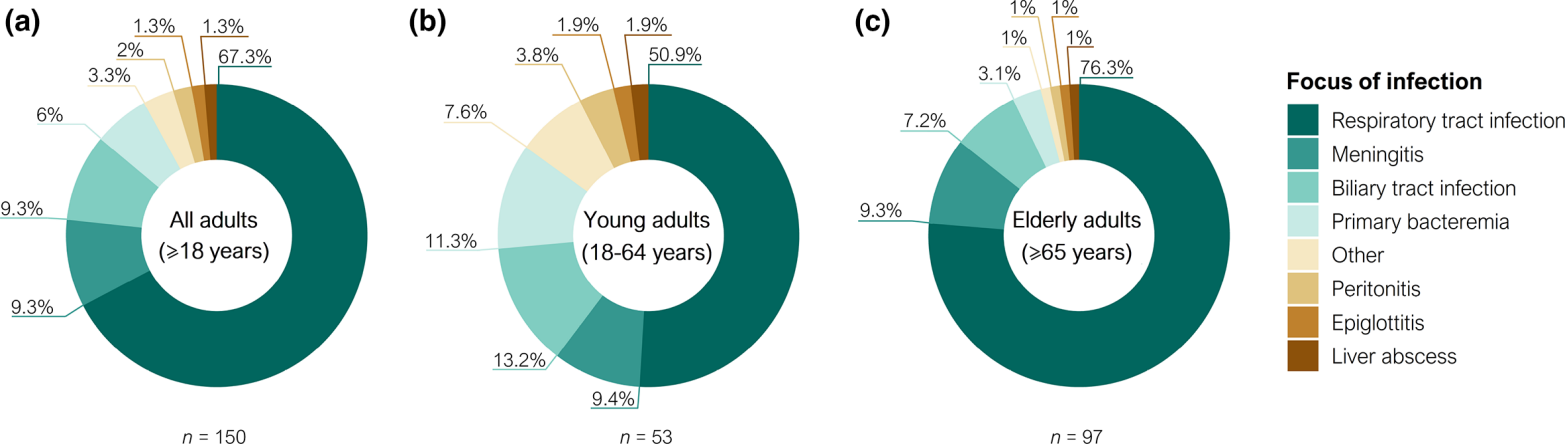
Frequencies of invasive *H. influenzae* infection by focus (2008–2019). (a) Focus of infection in the overall adult population. (b) Focus of infection in the young adult population (18–64 years). (c) Focus of infection in the elderly adult population (≥65 years). Infections caused by only one isolate were grouped as other: facial cellulitis, endometritis, urinary tract infection, and surgical wound infection (corresponding to young adults) and septic shock of an aortic valve prosthetic origin (corresponding to elderly adults).

Table 1 shows the demographics, clinical characteristics and underlying conditions of the patients. The most common sources of infection were respiratory tract infections (67.4 %), followed by meningitis and biliary tract infections (9.3 %, each) (Fig. 1a). Although respiratory infections were common in the entire population, they were significantly more frequent in the elderly than in young adults (76.3 and 50.9 %, respectively) (Fig. 1b, c). No statistically significant differences in the focus of infection were observed between the two periods.

**Table 1.** Demographic data, clinical characteristics and underlying conditions of patients with invasive *H. influenzae* disease

	Overall study period (2008–2019) *n*=150				Comparison between periods		
		Young adults (<65 years) *n*=53	Elderly adults (≥65 years) *n*=97	*P*-value	First period (2008–2013) *n*=82	Second period (2014–2019) *n*=68	*P*-value
**Characteristics [no. (%)]**							
Age (mean±sd; range)	67.5±14.9; 21–96	51.6±11.4; 21–64	76.1±7.7; 65–96	**<0.0001**	64.3±16.1; 21–96	71.2±12.3; 37–93	**0.0043**
Male sex	88 (58.7)	30 (56.6)	58 (59.8)	0.7311	52 (63.4)	36 (52.9)	0.2439
**Underlying conditions**							
Immunosuppressive therapy	60 (40.0)	22 (41.5)	38 (39.2)	0.86	28 (34.1)	32 (47.1)	0.13
Solid organ malignancy	54 (36.0)	17 (32.1)	37 (38.1)	0.48	26 (31.7)	28 (41.2)	0.24
Diabetes	39 (26.0)	8 (15.1)	31 (32.0)	**0.03**	18 (22.0)	21 (30.9)	0.26
Heart disease	40 (26.7)	7 (13.2)	33 (34.0)	**0.01**	15 (18.3)	25 (36.8)	**0.02**
COPD	30 (20.0)	4 (7.5)	26 (26.8)	**0.01**	18 (22.0)	12 (17.6)	0.54
Chronic liver disease	18 (12.0)	10 (18.9)	8 (8.2)	0.07	11 (13.4)	7 (10.3)	0.62
Hematologic malignancy*	16 (10.7)	7 (13.2)	9 (9.3)	0.58	9 (11.0)	7 (10.3)	1.00
Cerebrovascular disease	7 (4.7)	0 (0.0)	7 (7.2)	0.05	5 (6.1)	2 (2.9)	0.46
Organ transplant†	10 (6.7)	6 (11.3)	4 (4.1)	0.17	4 (4.9)	6 (8.8)	0.51
HIV	3 (2.0)	3 (5.7)	0 (0.0)	**0.04**	2 (2.4)	1 (1.5)	1.00
No underlying conditions	27 (18.0)	15 (28.3)	12 (12.4)	**0.02**	18 (22.0)	9 (13.2)	0.20
**Acquisition**							
Community acquired	130 (86.7)	43 (81.1)	87 (89.7)	0.21	71 (86.6)	59 (86.8)	1.00
Nosocomial	20 (13.3)	10 (18.9)	10 (10.3)	0.21	11 (13.4)	9 (13.2)	1.00
**Source of infection**							
Respiratory tract infection	101 (67.3)	27 (50.9)	74 (76.3)	**0.01**	49 (59.8)	52 (76.5)	**0.04**
Meningitis	14 (9.3)	5 (9.4)	9 (9.3)	1.00	9 (11.0)	5 (7.4)	0.58
Biliary tract infection	14 (9.3)	7 (13.2)	7 (7.2)	0.25	9 (11.0)	5 (7.4)	0.58
Primary bacteremia	9 (6.0)	6 (11.3)	3 (3.1)	0.07	7 (8.5)	2 (2.9)	0.18
Peritonitis	3 (2.0)	2 (3.8)	1 (1.0)	0.28	2 (2.4)	1 (1.5)	1.00
Other‡	9 (6.0)	6 (11.3)	3(3.1)	0.07	6 (7.3)	3 (4.4)	0.51
**Charlson comorbidity index**	4.8±2.5	3.4±3.0	5.5±1.8	**<0.0001**	4.3±2.4	5.3±2.5	**0.01**
**30 day mortality**	27 (18.0)	10 (18.9)	17 (17.5)	0.83	17 (20.7)	10 (14.7)	0.40

*Myeloma (*n*=8, 5.3%), leukaemia and lymphoma (*n*=4, 2.7 % each).

†Liver (*n*=4, 2.7%), bone marrow (*n*=3, 2.0%), kidney (*n*=2, 1.3%) and heart (*n*=1, 0.7%).

‡Epiglotittis, liver abscess (*n*=2, 1.3 % each), endometritis, facial cellulites, septic shock of an aortic valve prosthetic origin, surgical wound infection, and urinary tract infection (*n*=1, 0.7 % each).

The most common comorbidities were immunosuppressive therapy (40.0%) and solid organ malignancies (36.0%), which were strongly associated with 30 day mortality. Diabetes, heart disease and COPD were more common in the elderly, while HIV was more frequent in younger adults. The only difference observed between the two periods was an increase in the number of patients with heart disease. The overall 30 day mortality was 18.0 % (*n*=27), which decreased between the periods (20.7 and 14.7 %, respectively) and did not differ between the age groups (18.9 and 17.5 %, respectively) (Table 1). The Charlson comorbidity index (CCI) was significantly higher in the elderly adults (5.5±1.8) compared to young adults (3.4±3.0) (*P*-value<0.0001). Nevertheless, 30 day mortality in young adults was associated with a higher CCI (5.2±3.8) compared to young adults who survived (3.0±2.7) (*P*-value=0.04), while no differences were observed in the elderly (5.9±1.6 and 5.4±1.8, respectively) (Table S2).

### Antimicrobial susceptibility and resistance determinants

Susceptibility rates for all the antibiotics tested were similar in the two periods (Table 2). All the strains were susceptible to amoxicillin/clavulanic acid, cefotaxime, ceftriaxone, imipenem, chloramphenicol and tetracycline. On the other hand, 37 strains (24.7 %) were resistant to cotrimoxazole, 19 (12.7 %) to ampicillin, 3 (2.0 %) to ciprofloxacin, and 1 (0.7 %) to cefuroxime, cefepime and azithromycin.

**Table 2.** Comparison of antimicrobial resistance rates of invasive *H. influenzae* during the two study periods

Antimicrobial compound	MIC breakpoints (CLSI)		First period*			Second period			*P*-value
			2008–2013 (*n*=70)			2014–2019 (*n*=68)			
	S ≤	R ≥	MIC_50_ (mg l^−1^)	MIC_90_ (mg l^−1^)	%R	MIC_50_ (mg l^−1^)	MIC_90_ (mg l^−1^)	%R	
Ampicillin	1	4	0.25	2	10	0.25	>4	17.6	0.22
Amoxicillin/clavulanic acid†	4	8	≤0.5	2	0	≤0.5	1	0	1
Cefuroxime	4	16	1	2	0	≤0.5	1	1.5	0.49
Cefepime	2	4	≤0.25	≤0.25	0	≤0.25	≤0.25	1.5	0.49
Cefotaxime	2	4	≤0.06	≤0.06	0	≤0.06	≤0.06	0	1
Ceftriaxone	2	4	≤0.12	≤0.12	0	≤0.12	≤0.12	0	1
Imipenem	4	8	0.5	1	0	≤0.12	0.25	0	1
Chloramphenicol	2	8	≤1	≤1	0	≤1	≤1	0	1
Tetracycline	2	8	≤1	2	0	≤1	≤1	0	1
Ciprofloxacin	1	2	≤0.03	≤0.03	0	≤0.03	≤0.03	4.4	0.12
Co-trimoxazole‡	0.5	4	≤0.5	>2	26	≤0.5	>2	27.9	0.85
Azithromycin	4	8	1	2	1.4	2	4	0	0.49

*Only 70 of the 82 strains of the first period were viable.

†The ratio of amoxicillin/clavulanic acid was 2 : 1.

‡The ratio of co-trimoxazole was 1 : 19.

MIC, Minimum inhibitory concentration; R, resistant; %R, resistance rate; S, susceptible.

During the second period (2014–2019), there was a slight increase in β-lactam resistance and an emergence of resistance to ciprofloxacin (Table 2). Twelve strains (17.6%) were resistant to ampicillin due to β-lactamase production: eleven NTHi strains had a *bla*_TEM-1_ gene and one serotype f strain carried a *bla*_ROB-1_ gene in a pB1000 plasmid (GU080063). The latter was also resistant to cefuroxime and cefepime. Amino acid substitutions in PBP3 were observed in 26 strains (38.2%) and were classified according to Dabernat *et al.* [25] (Table 3). Eleven strains were classified in group II, characterized by the K526N substitution, and showed reduced susceptibility to ampicillin. Fluoroquinolone resistance was observed in three strains (4.4%), which, despite being low, indicated the emergence of resistance to fluoroquinolones since no ciprofloxacin-resistant strains were identified in the previous period. Resistance was associated with changes in GyrA (S84L and D88G in two of the strains, and S84L and D88N in one strain) and ParC (S84I in all the ciprofloxacin-resistant strains) (Dataset S1).

**Table 3.** Mechanisms of β-lactam antimicrobial resistance among invasive *H. influenzae* strains (2014–2019). Amino acid substitutions in PBP3 and classification according to Dabernat *et al.* [25], and β-lactamase production

	No. isolates	MIC (mg l^−1^)		Amino acid substitutions										β-lactamase*	
		AMP	AMC†	Ile	Asp	Ala	Met	Ala	Ile	Gly	Ala	Asn	Ala	*bla*_TEM-1_	*bla*_ROB-1_
				348	350	368	377	437	449	490	502	526	530		
No changes	32	≤0.12–0.25	≤0.5											−	−
	10	4 ->4	≤0.5–2											+	−
Group IIa	1	0.5	1									Lys	Ser	−	−
	1	1	1		Asn					Glu		Lys	Ser	−	−
	1	1	1		Asn							Lys		−	−
Group IIb	1	1	1							Glu	Val	Lys		−	−
	2	0.5–1	1		Asn		Ile				Val	Lys		−	−
Group IIc	2	1	1								Thr	Lys		−	−
	2	1	1		Asn						Thr	Lys		−	−
Group IId	1	1	1						Val			Lys		−	−
Miscellaneous	10	0.25–0.5	≤0.5–1		Asn									−	−
	1	≤0.12	≤0.5	Val										−	−
	1	>4	2		Asn									−	+
	1	>4	≤0.5		Asn									+	−
	1	≤0.12	≤0.5		Asn			Ser						−	−
	1	0.25	≤0.5			Thr								−	−

*β-lactamase production: positive (+), negative (−).

†The ratio of amoxicillin/clavulanic acid was 2 : 1.

AMC, amoxicillin/clavulanic acid; AMP, ampicillin; MIC, Minimum inhibitory concentration.

### Serotype distribution and molecular epidemiology

**Fig. 2.**
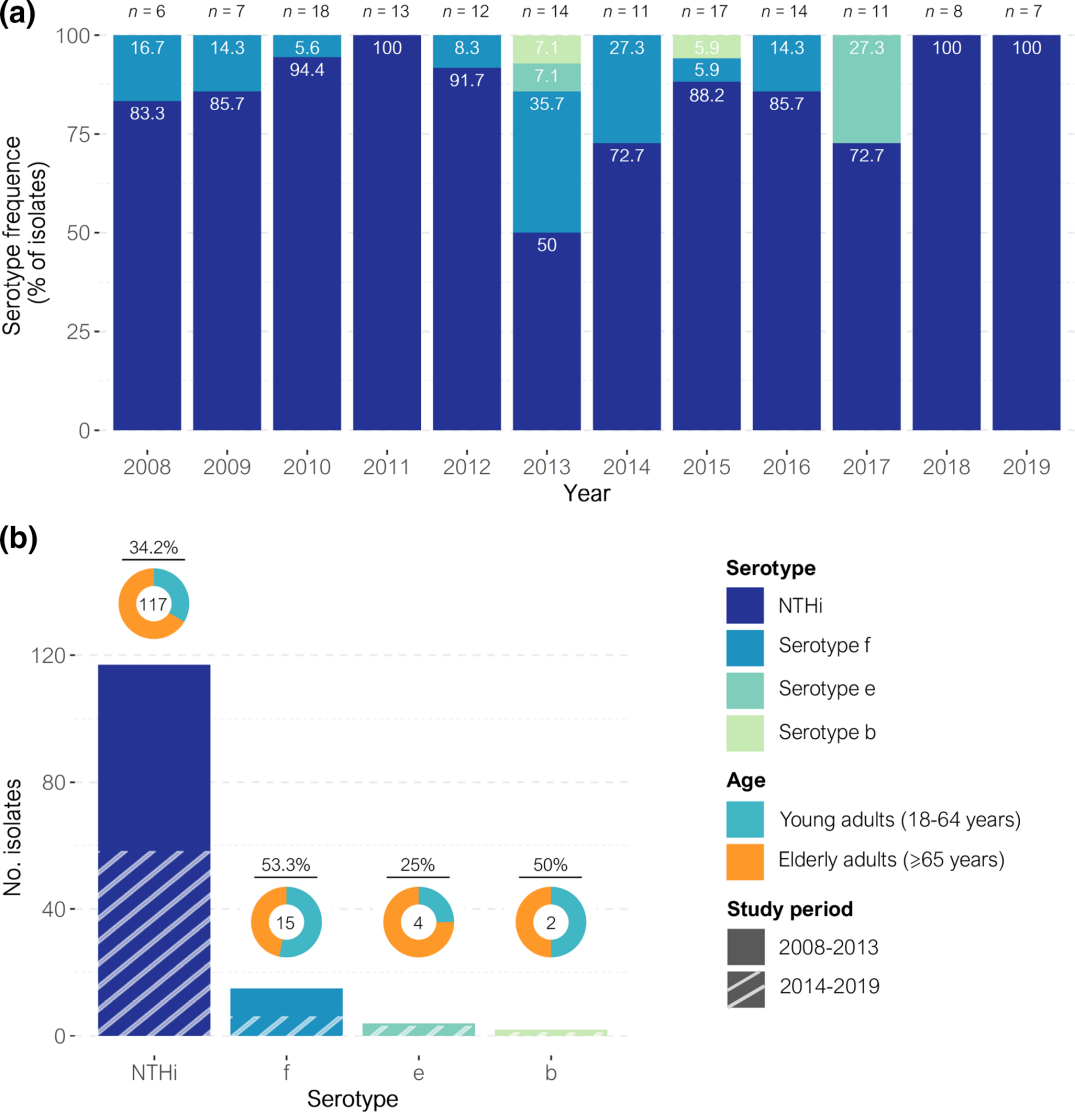
Distribution of *H. influenzae* serotypes in invasive disease (2008–2019). (a) Frequencies of *H. influenzae* serotypes causing invasive disease per year. Frequencies are displayed at the upper part of each bar. (b) Distribution of *H. influenzae* serotypes causing invasive disease between 2008 and 2019. Donut charts, at the top of each bar, show the age group frequency; the frequency (%) in young adults is shown above. The number of isolates presenting each serotype is shown inside the donut charts.

NTHi was the main cause of invasive disease in both periods in contrast to the overall low percentage of capsulated strains, mainly serotype f and serotype e, both of which had disappeared at the end of the study (Fig. 2a). Serotype b maintained a low frequency, with only one case detected in each period. Overall, 117 cases were caused by NTHi (*n*=59 in the first period and *n*=58 in the second period), 15 were caused by serotype f (*n*=9 and *n*=6, respectively), four were due to serotype e (*n*=1 and *n*=3, respectively), and two were due to serotype b strains (*n*=1 and *n*=1, respectively) (Fig. 2b). The remaining 12 strains from the first period were not available for microbiological analysis. There were no differences in serotype distribution by age group nor by study period.

MLST and PHYLOViZ analysis showed high genetic heterogeneity, with 84 different STs identified among the 117 NTHi strains. The most common in the first period was ST57 (*n*=3), while ST103 was the most prevalent in the second period (*n*=4), followed by ST3 and ST160 (*n*=3, each). Two of the most common STs, ST103 and ST160, produced a TEM-1 β-lactamase, and both of them showed increased prevalence from the previous period: ST103 from one to four isolates, while ST160, which was not detected in the first period, caused three cases of invasive disease in the second period. Despite the great variability among NTHi, PHYLOViZ revealed two clusters, which each consisted of clones that were linked to one another by sharing at least five MLST loci with another clone in the group (Fig. S1). One of the clusters was related to ST3 and included 13 STs (*n*=20), while the other was associated with ST103 and included five STs (*n*=10). Moreover, there was an increase in the number of isolates belonging to these clusters, with 13 in the first period (ten associated with the ST3 cluster and three with the ST103 cluster) and 17 in the second period (ten and seven, respectively). On the other hand, capsulated strains showed high clonality, with a few related STs (single-locus variants, SLVs) identified for each serotype in both periods: ST124 (*n*=12) and ST2354, ST2361, and ST2366 (*n*=1, each) for serotype f; ST18 and ST386 (*n*=2, each) for serotype e; and ST6 and ST2364 (*n*=1, each) for serotype b. No differences in ST distribution were observed in the capsulated strains between the periods.

### Phylogeny and the NTHi population structure

In the first study period [15], only the seven MLST genes were sequenced. However, in the second period, due to improved methodological techniques, all invasive *H. influenzae* genomes were sequenced through WGS.

**Fig. 3.**
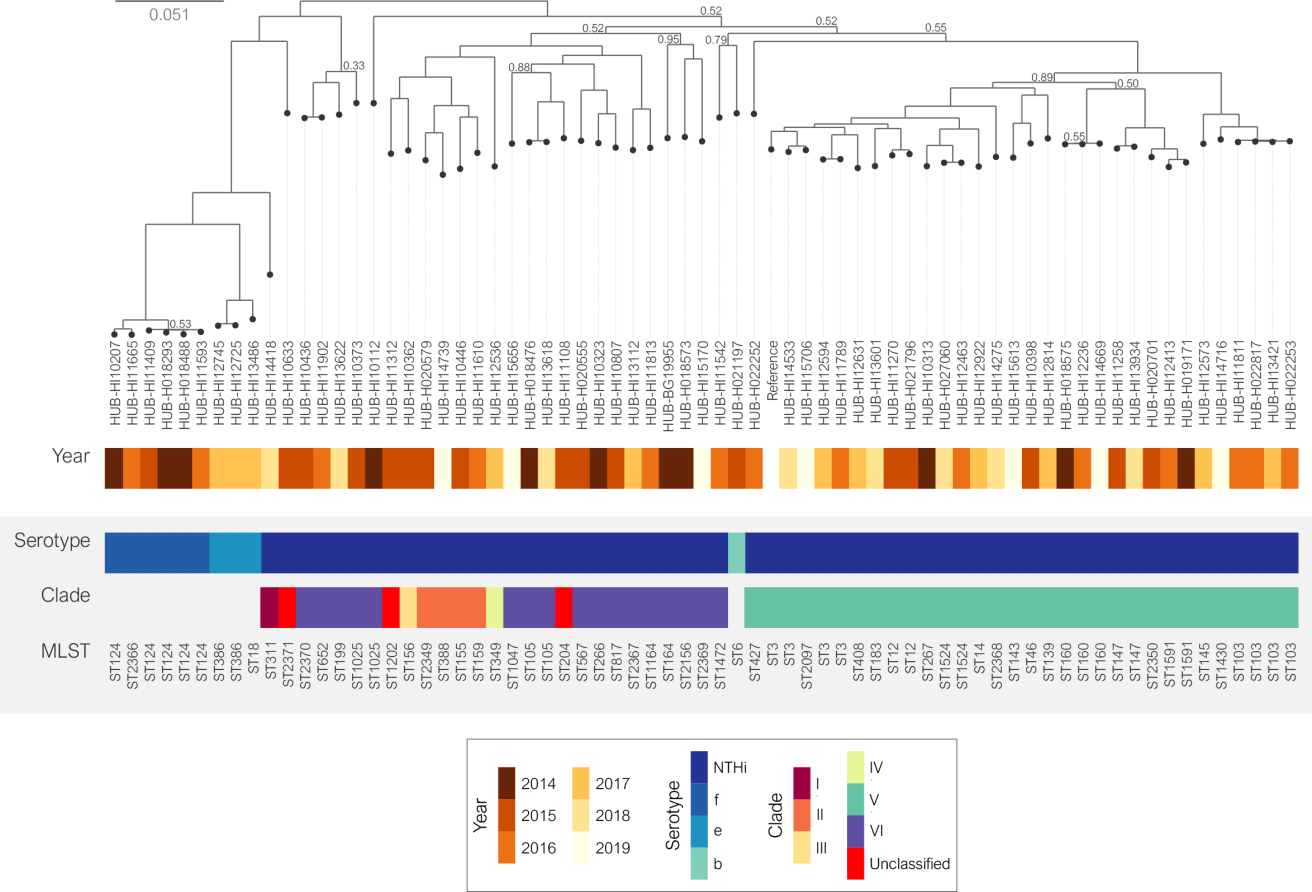
Core-SNP phylogenetic tree of invasive *H. influenzae* isolates (2014–2019). NTHi was classified into clades (I–VI) as defined by De Chiara *et al.* [12]. Relative bootstrap values other than one are given at branch nodes. Relative bootstrap values below 0.75 indicate that the branches are poorly supported. Strain Hi375 (CP009610) was used as the reference.

**Fig. 4.**
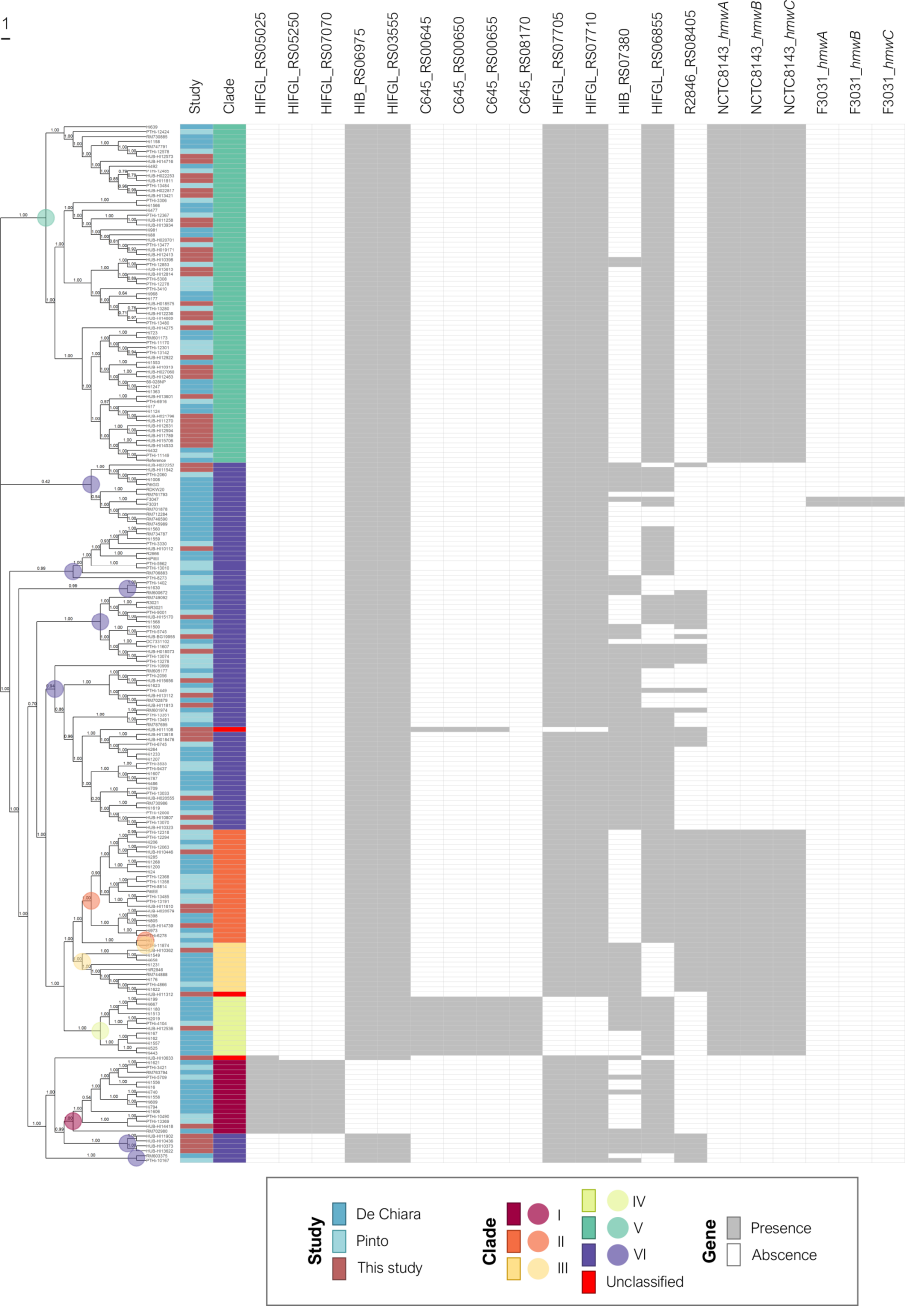
Assembly-based core-SNP phylogenetic tree and clade distribution of nontypeable *H. influenzae *. The tree includes 213 NTHi genomes from three studies: De Chiara *et al.* [12], Pinto *et al.* [14] and Carrera-Salinas *et al.* (this study). Genomes were distributed in different clades (I–VI), as previously proposed according to the presence or absence of 17 accessory genes [12, 14]: HIFGL_RS05025: *dinB* (DNA polymerase IV); HIFGL_RS05250: ‘YbhB/YbcL family Raf kinase inhibitor-like protein’; HIFGL_RS07070: ‘ABC transporter substrate-binding protein’; HIB_RS06975: ‘7-carboxy-7-deazaguanine synthase QueE’; HIFGL_RS03555: ‘DEAD/DEAH box helicase family protein’; C645_RS00645: ‘Hypothetical protein’; C645_RS00650: ‘pirin family protein’; C645_RS00655: ‘DUF1016 family protein’; C645_RS08170: ‘Hypothetical protein’; HIFGL_RS07705: ‘nucleotidyltransferase domain-containing protein’; HIFGL_RS07710: ‘nucleotidyltransferase substrate-binding subunit’; HIB_RS07380: ‘ABC transporter ATP-binding protein’; HIFGL_RS06855: ‘5-oxoprolinase/urea amidolyase family protein’; R2846_RS08405: ‘TonB-dependent receptor’; NCTC8143_*hmwA/B/C* and F3031_*hmwA/B/C: hmwA/B/C* (high molecular weight protein A, B or C). The coloured dots show the most recent common ancestor (MRCA) found in each clade. Relative bootstrap values are given at branch nodes (values below 0.75 are poorly supported).

**Fig. 5.**
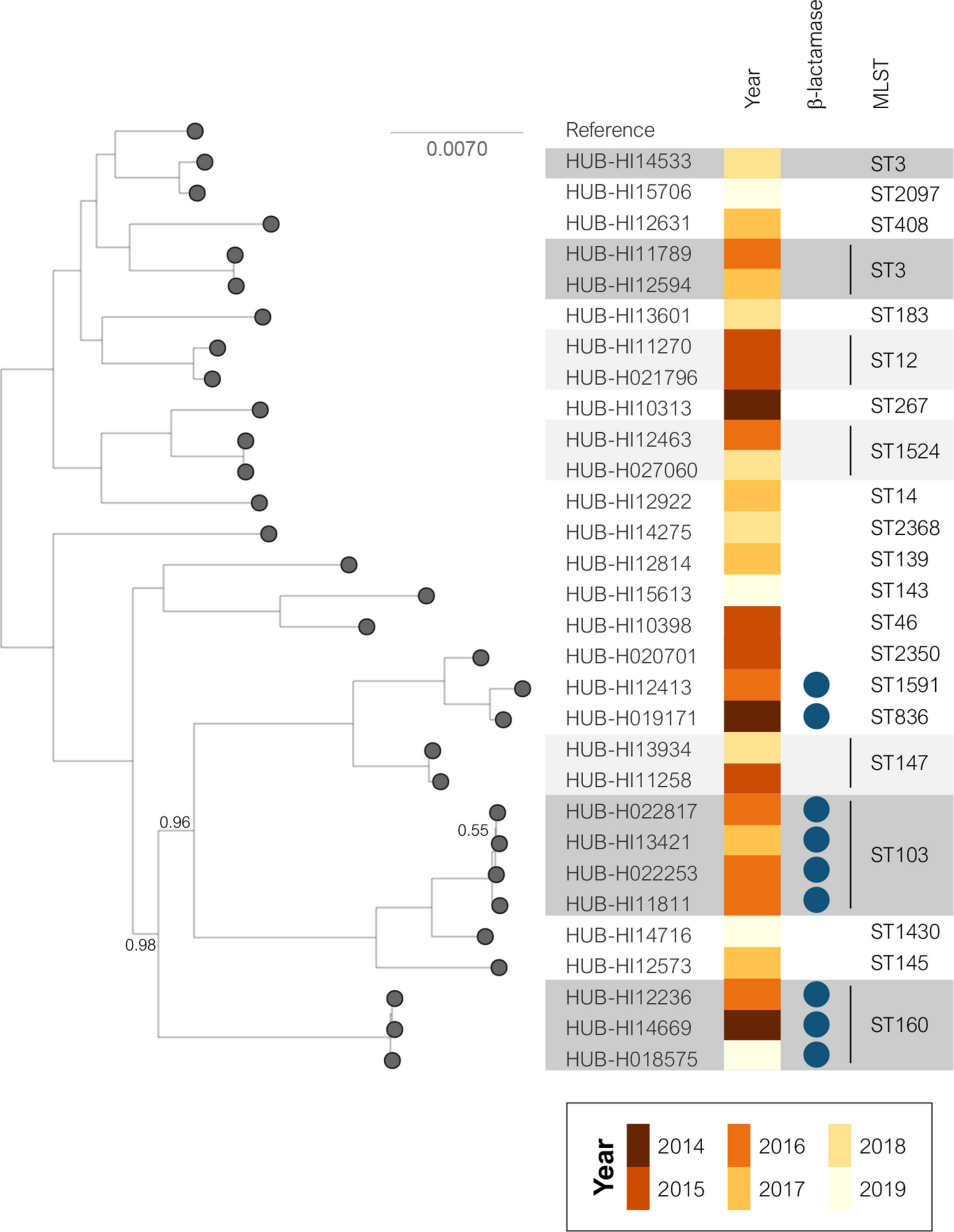
Phylogenetic tree of invasive NTHi isolates from clade V (2014–2019). Blue circles indicate the presence of a TEM-1 β-lactamase. Shadowed areas highlight the most common STs (dark grey for ≥3 isolates and light grey for two isolates included in the ST). Relative bootstrap values other than one are given at branch nodes. Relative bootstrap values below 0.75 indicate that the branches are poorly supported. Strain Hi375 was used as the reference.

The MLST results were confirmed by the phylogenetic analysis (Fig. 3): 28 NTHi strains showed high genetic diversity, while the remaining 30 strains, most of which were clustered by MLST, shared the same monophyletic origin. The invasive NTHi isolates were classified into clades based on the presence or absence of 17 accessory genes as previously described [12, 14]. To provide a context for the invasive NTHi population, we combined our genome collection with the 155 NTHi assemblies selected for clade definition [12, 14] (Fig. 4) . This classification showed that most of the NTHi strains belonged to clades V and VI (32.5 and 37.7 %, respectively). Three strains belonging to ST204, ST1202 and ST2371 could not be categorized into any of the clades. According to their monophyletic origins, the ST204 and ST1202 strains were related to clade VI and III, respectively, while ST2371 had a phylogenetic origin unrelated to the other clades (Fig. 3). The SNP-based phylogenetic analysis revealed high genetic similarity between the clade V isolates, which included the 17 strains belonging to the large MLST clusters formed by the most prevalent STs and the strains belonging to ST160 (*n*=3), ST12, ST147 and ST1524 (*n*=2, each), and ST183, ST836, ST1591 and ST2350 (*n*=1, each). Moreover, nine of the 12 β-lactamase producing strains were included in this clade (Figs 3 and 5).

In addition, each clade constituted a different monophyletic lineage, except for clades II, III and VI, in which the strains showed more diverse phylogenetic origins (Fig. 4). The recombination analyses revealed that a large proportion of sites were affected by recombination, especially in NTHi strains (*r*/*m*=7.85, sd=12.42) compared to capsulated isolates (*r*/*m*=1.14, sd=1.38) (Figs S2 and S3).

## Discussion

Invasive *H. influenzae* disease is uncommon, but can be severe, especially in adults with comorbidities. *H. influenzae* is a matter of concern due to its community-acquired burden and the high prevalence of ampicillin resistance it presents [26]. For these reasons, surveillance has become crucial for the control of *H. influenzae *. In this study, we provide an update on the epidemiology of invasive *H. influenzae* disease over a 12 year period and analysed the population structure of NTHi using a clade-related classification.

Between 2008 and 2019, the overall incidence of invasive disease in Bellvitge University Hospital was 2.07/100 000, which is consistent with the incidence rate found in Europe and the USA [27, 28]. Invasive disease had a higher incidence in individuals over 65 years of age, in line with the underlying conditions that are more common in older adults such as COPD, heart disease and diabetes [8, 29, 30]. By contrast, young adults presented more cases of invasive disease due to unusual origins, such as peritonitis, liver abscess, endometritis, facial cellulitis or urinary tract infection, which, although less common, typically affect immunocompetent young adults [31–33]. The overall 30 day mortality was 18.0 %, within the range of 10–20 % found in other studies [34–36]. It should be noted that the 30 day mortality decreased between the two periods (20.7 and 14.7 %, respectively), probably due to a better diagnosis of bloodstream infections and a more efficient clinical management of these patients. Moreover, severe underlying conditions were the main cause of mortality, especially in young adults.

Invasive *H. influenzae* infections caused by NTHi and non-type-b serotypes have gradually increased in Europe between 2000 and 2014 [8]. Our study showed a prevalence of 84.8 % for NTHi, 10.9 % for serotype f, 2.9 % for serotype e and 1.4 % for serotype b. Likewise, other studies have reported that NTHi strains are the most frequent cause of invasive disease, ranging from 54.6–82 % of the cases [27, 36–38], while the frequency of the capsulated serotypes varies according to the geographical region. The results reported to the ECDC [39] indicated that serotype b isolates caused 7 % of invasive infections, showing a marked reduction since the introduction of the serotype b vaccination, while prevalence of serotype f, which caused 9 % of invasive infections, was increasing annually. However, in some countries such as Italy and Portugal [9, 38], serotype b caused more cases of invasive disease than serotype f despite high vaccine coverage, highlighting the importance of epidemiological surveillance. Although rare in the pre-vaccine era, serotype a is currently emerging as a cause of serious morbidity and mortality in Canada, USA, Brazil and Australia [40], causing up to 23.1 % of invasive infections in Canada [37]. Langereis *et al.* [34] observed that most of the infections caused by this serotype occurred in children, probably explaining why it was not detected in our study since it only included the adult population.

Antimicrobial resistance rates remained constant between the two periods of our study, with only a slight increase in β-lactam and fluoroquinolone resistance. TEM-1 β-lactamase was the most common cause of resistance to ampicillin, while one isolate carried a plasmid with the ROB-1 enzyme. San Millan *et al.* [41] demonstrated that the presence of the pB1000 plasmid carrying the ROB-1 β-lactamase reduced the fitness of *H. influenzae *, which could explain the low prevalence of this β-lactamase type. Despite being low, fluoroquinolone resistance emerged in the second period. Fluoroquinolones are commonly used to treat respiratory infections, which may lead to resistance acquisition. Although the fluoroquinolone resistance rate is low worldwide [42, 43], vigilance is required to monitor its progression.

NTHi had higher genetic variability and a larger proportion of sites affected by recombination compared to capsulated isolates or other bacterial species [44]. Most NTHi are composed of individual STs, even if some strains may be grouped occasionally into clonal complexes [9, 38]. SNP-based phylogenetic analysis together with clade-based classification [12, 14] could be useful in identifying NTHi subpopulations with specific pathogenesis and epidemiology. More than half of our invasive NTHi strains were grouped into the same cluster by PHYLOViZ, SNP-based phylogenetic analysis or clade-based classification. All these NTHi isolates were clustered into clade V and belonged to the most prevalent STs (ST3, ST14, ST103, and ST139). They shared a common phylogenetic origin with the clones identified in the first period, and some of them showed increased frequency over time. In addition, in clade V, we found 9 of the 12 invasive isolates with β-lactamase activity, which were associated mainly with ST103, ST160, ST836 and ST1591. Some studies have already reported that ST103 and ST160 are among the most frequent invasive isolates [9, 38]. It should therefore be considered whether the increase in β-lactam resistance is due to the rise of these clones or whether β-lactamase production gives an advantage over the other less frequent clones. In any case, the evolution of the clade V clones should be monitored.

The second most common NTHi clade was VI followed by clade II, while clades I, III and IV were rarely observed. Isolates belonging to clades II and III showed high genetic variability, which could be solved by classification into subclades defined by their different monophyletic origins. On the other hand, clade VI isolates had different monophyletic origins, despite sharing the pattern of accessory genes described by Pinto *et al.* [14]. The inclusion of more samples and the consideration of other accessory genome genes in the analysis may improve this clade classification. Based on their clinical characteristics, it was difficult to differentiate NTHi isolates from among each clade since they were clinically diverse, which is consistent with other studies showing no correlation between the phylogenetic classification and clinical or geographical origins [12, 45]. However, Kc *et al.* [46] classified NTHi isolates into eight clades in which, despite having COPD strains distributed among all the clades, they could differentiate between COPD and non-COPD strains according to the composition of the accessory genome. This suggests that further studies are needed to improve the classification of NTHi strains and to find an association between the clades, the composition of the accessory genome, clinical data and pathogenesis.

Although MLST is useful for classifying capsulated isolates and it did cluster some of the NTHi isolates, it is not the most appropriate technique to study the heterogeneous NTHi population. Details of the heterogeneity of the *H. influenzae* population can be missed if only MLST is performed, particularly if the sample size is small and the analysed clones do not belong to the most prevalent STs. For this reason, we suggest that the combination of phylogenetic analysis and clade-related classification based on the accessory genome is a good approach to monitor the evolution of invasive *H. influenzae* clones and to control the rise of clones associated with β-lactamase production. Further studies will elucidate clades with high phylogenetic variability.

In conclusion, invasive *H. influenzae* disease is mainly associated with NTHi and its incidence increases with age, especially in patients with underlying disease. Serotype f remains the most common among the capsulated strains, followed by serotype e, although both disappeared by the end of the study. Continued surveillance of invasive *H. influenzae* disease is necessary to control the emergence of NTHi and non-vaccine serotype isolates, as well as the rising resistance to β-lactams and fluoroquinolones. We propose that the NTHi population structure should be studied through phylogenetic analysis and clade-related classification, as it clusters the main STs associated with invasive disease. This is an alternative to overcome the limitations of MLST and to comprehend the genetic diversity and dynamics of NTHi.

## Supplementary Data

Supplementary material 1

Supplementary material 2

Supplementary material 3

Supplementary material 4

## References

[1] LaCross NC, Marrs CF, Gilsdorf JR (2013). Population structure in nontypeable *Haemophilus influenzae*. Infect Genet Evol.

[2] Slack MPE (2015). A review of the role of *Haemophilus influenzae* in community-acquired pneumonia. Pneumonia (Nathan).

[3] Nørskov-Lauritsen N (2014). Classification, identification, and clinical significance of *Haemophilus* and *Aggregatibacter* species with host specificity for humans. Clin Microbiol Rev.

[4] Agrawal A, Murphy TF (2011). *Haemophilus influenzae* infections in the *H. influenzae* type b conjugate vaccine era. J Clin Microbiol.

[5] Sethi S, Murphy TF (2008). Infection in the pathogenesis and course of chronic obstructive pulmonary disease. N Engl J Med.

[6] Cardines R, Giufrè M, Pompilio A, Fiscarelli E, Ricciotti G (2012). *Haemophilus influenzae* in children with cystic fibrosis: Antimicrobial susceptibility, molecular epidemiology, distribution of adhesins and biofilm formation. Int J Med Microbiol.

[7] van de Beek D, Brouwer M, Hasbun R, Koedel U, Whitney CG (2016). Community-acquired bacterial meningitis. Nat Rev Dis Primers.

[8] Wang S, Tafalla M, Hanssens L, Dolhain J (2017). A review of *Haemophilus influenzae* disease in Europe from 2000-2014: challenges, successes and the contribution of hexavalent combination vaccines. Expert Rev Vaccines.

[9] Heliodoro CIM, Bettencourt CR, Bajanca-Lavado MP, Portuguese Group for the Study of Haemophilus influenzae invasive infection (2020). Molecular epidemiology of invasive *Haemophilus influenzae* disease in Portugal: an update of the post-vaccine period, 2011-2018. Eur J Clin Microbiol Infect Dis.

[10] Wen S, Feng D, Chen D, Yang L, Xu Z (2020). Molecular epidemiology and evolution of *Haemophilus influenzae*. Infect Genet Evol.

[11] Robinson DA, Thomas JC, Hanage WP (2011). Genetics and Evolution of Infectious Diseases.

[12] De Chiara M, Hood D, Muzzi A, Pickard DJ, Perkins T (2014). Genome sequencing of disease and carriage isolates of nontypeable *Haemophilus influenzae* identifies discrete population structure. Proc Natl Acad Sci U S A.

[13] Staples M, Graham RMA, Jennison AV (2017). Characterisation of invasive clinical *Haemophilus influenzae* isolates in Queensland, Australia using whole-genome sequencing. Epidemiol Infect.

[14] Pinto M, González-Díaz A, Machado MP, Duarte S, Vieira L (2019). Insights into the population structure and pan-genome of *Haemophilus influenzae*. Infect Genet Evol.

[15] Puig C, Grau I, Marti S, Tubau F, Calatayud L (2014). Clinical and molecular epidemiology of *Haemophilus influenzae* causing invasive disease in adult patients. PLoS One.

[16] Clinical Laboratory Standards Institute (2019). CLSI Supplement Document M100.

[17] Francisco AP, Vaz C, Monteiro PT, Melo-Cristino J, Ramirez M (2012). PHYLOViZ: Phylogenetic inference and data visualization for sequence based typing methods. BMC Bioinformatics.

[18] Watts SC, Holt KE (2019). HICAP: In silico serotyping of the Haemophilus influenzae capsule locus. J Clin Microbiol.

[19] Bortolaia V, Kaas RS, Ruppe E, Roberts MC, Schwarz S (2020). ResFinder 4.0 for predictions of phenotypes from genotypes. J Antimicrob Chemother.

[20] Kozlov AM, Darriba D, Flouri T, Morel B, Stamatakis A (2019). RAxML-NG: a fast, scalable and user-friendly tool for maximum likelihood phylogenetic inference. Bioinformatics.

[21] Pattengale ND, Alipour M, Bininda-Emonds ORP, Moret BME, Stamatakis A (2010). How many bootstrap replicates are necessary?. J Comput Biol.

[22] Croucher NJ, Page AJ, Connor TR, Delaney AJ, Keane JA (2015). Rapid phylogenetic analysis of large samples of recombinant bacterial whole genome sequences using Gubbins. Nucleic Acids Res.

[23] Treangen TJ, Ondov BD, Koren S, Phillippy AM (2014). The Harvest suite for rapid core-genome alignment and visualization of thousands of intraspecific microbial genomes. Genome Biol.

[24] Yu G, Smith DK, Zhu H, Guan Y, Lam TT (2016). ggtree : an r package for visualization and annotation of phylogenetic trees with their covariates and other associated data. Methods Ecol Evol.

[25] Dabernat H, Delmas C, Seguy M, Pelissier R, Faucon G (2002). Diversity of beta-lactam resistance-conferring amino acid substitutions in penicillin-binding protein 3 of *Haemophilus influenzae*. Antimicrob Agents Chemother.

[26] Tacconelli E, Carrara E, Savoldi A, Harbarth S, Mendelson M (2018). Discovery, research, and development of new antibiotics: the WHO priority list of antibiotic-resistant bacteria and tuberculosis. Lancet Infect Dis.

[27] Whittaker R, Economopoulou A, Dias JG, Bancroft E, Ramliden M (2017). Epidemiology of Invasive *Haemophilus influenzae* Disease, Europe, 2007-2014. Emerg Infect Dis.

[28] Soeters HM, Blain A, Pondo T, Doman B, Farley MM (2018). Current epidemiology and trends in invasive *Haemophilus influenzae* Disease-United States, 2009-2015. Clin Infect Dis.

[29] GBD Chronic Respiratory Disease Collaborators (2020). Prevalence and attributable health burden of chronic respiratory diseases, 1990-2017: a systematic analysis for the Global Burden of Disease Study 2017. Lancet Respir Med.

[30] Haro JM, Tyrovolas S, Garin N, Diaz-Torne C, Carmona L (2014). The burden of disease in Spain: results from the global burden of disease study 2010. BMC Med.

[31] Fujii M, Gomi H, Ishioka H, Takamura N (2017). Bacteremic renal stone-associated urinary tract infection caused by nontypable *Haemophilus influenzae*: A rare invasive disease in an immunocompetent patient. IDCases.

[32] Stærk M, Tolouee SA, Christensen JJ (2018). Nontypable *Haemophilus influenzae* septicemia and urinary tract infection associated with renal stone disease. Open Microbiol J.

[33] Martin D, Dbouk RH, Deleon-Carnes M, del Rio C, Guarner J (2013). *Haemophilus influenzae* acute endometritis with bacteremia: Case report and literature review. Diagn Microbiol Infect Dis.

[34] Langereis JD, de Jonge MI (2015). Invasive disease caused by nontypeable *Haemophilus influenzae*. Emerg Infect Dis.

[35] Chiappini E, Inturrisi F, Orlandini E, de Martino M, de Waure C (2018). Hospitalization rates and outcome of invasive bacterial vaccine-preventable diseases in Tuscany: A historical cohort study of the 2000-2016 period. BMC Infect Dis.

[36] Blain A, MacNeil J, Wang X, Bennett N, Farley MM (2014). Invasive *Haemophilus influenzae* Disease in Adults ≥65 Years, United States, 2011. Open Forum Infect Dis.

[37] Tsang RSW, Shuel M, Whyte K, Hoang L, Tyrrell G (2017). Antibiotic susceptibility and molecular analysis of invasive *Haemophilus influenzae* in Canada, 2007 to 2014. J Antimicrob Chemother.

[38] Giufrè M, Fabiani M, Cardines R, Riccardo F, Caporali MG (2018). Increasing trend in invasive non-typeable *Haemophilus influenzae* disease and molecular characterization of the isolates, Italy, 2012-2016. Vaccine.

[39] European Centre for Disease Prevention and Control (ECDC) (2020). Haemophilus influenzae annual epidemiological report for 2018.

[40] Ulanova M, Tsang RSW (2014). *Haemophilus influenzae* serotype a as a cause of serious invasive infections. Lancet Infect Dis.

[41] San Millan A, Garcia-Cobos S, Escudero JA, Hidalgo L, Gutierrez B (2010). *Haemophilus influenzae* clinical isolates with plasmid pB1000 bearing blaROB-1: Fitness cost and interspecies dissemination. Antimicrob Agents Chemother.

[42] Shoji H, Shirakura T, Fukuchi K, Takuma T, Hanaki H (2014). A molecular analysis of quinolone-resistant *Haemophilus influenzae*: Validation of the mutations in quinolone resistance-determining regions. J Infect Chemother.

[43] Puig C, Tirado-Vélez JM, Calatayud L, Tubau F, Garmendia J (2015). Molecular characterization of fluoroquinolone resistance in nontypeable *Haemophilus influenzae* clinical isolates. Antimicrob Agents Chemother.

[44] Vos M, Didelot X (2009). A comparison of homologous recombination rates in bacteria and archaea. ISME J.

[45] Pettigrew MM, Ahearn CP, Gent JF, Kong Y, Gallo MC (2018). *Haemophilus influenzae* genome evolution during persistence in the human airways in chronic obstructive pulmonary disease. Proc Natl Acad Sci U S A.

[46] Kc R, Leong KWC, Harkness NM, Lachowicz J, Gautam SS (2020). Whole-genome analyses reveal gene content differences between nontypeable *Haemophilus influenzae* isolates from chronic obstructive pulmonary disease compared to other clinical phenotypes. Microbial Genomics.

