# Supplementary material for: Epidemiology and population structure of Haemophilus influenzae causing invasive disease
Source: Microb Genom. 2021 Dec 13;7(12):000723. doi: 10.1099/mgen.0.000723 (PMC8767337; doi:10.1099/mgen.0.000723)
Supplement: Supplementary material 1 [file mgen-7-0723-s001.pdf]

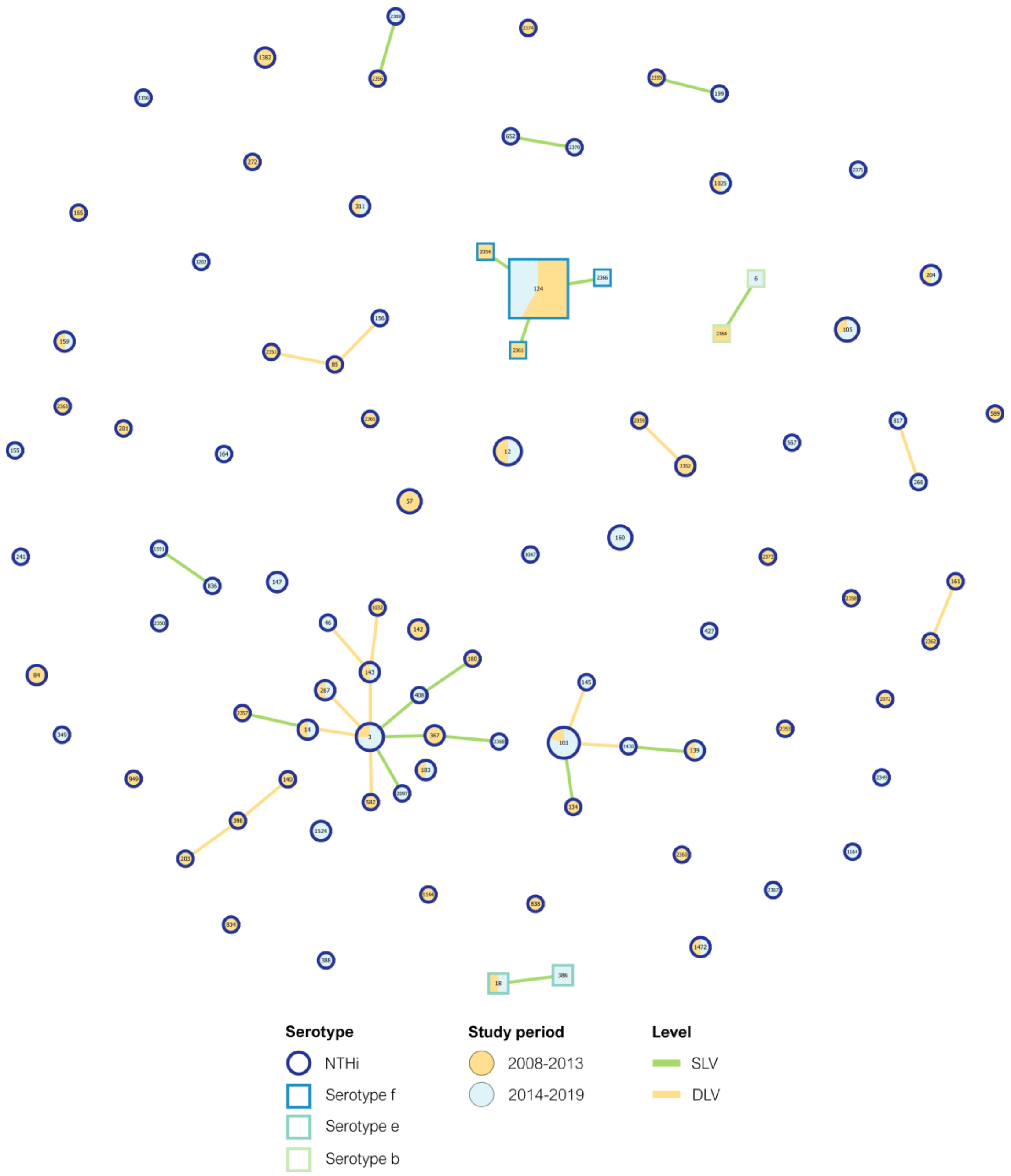

Figure S1. Relationship between the invasive *H. influenzae* isolates defined by PHYLOViZ. Circles for NTHi clones and squares for capsulated *H. influenzae* strains correspond to unique STs. The size of the circles/squares represents the number of isolates belonging to each ST. The inner color indicates the proportion of isolates for each period (2008-2013 [15] and 2014-2019 (this study)), while the outline color indicates the serotype. The relationship between the STs is defined as single- (SLV) or double-locus (DLV) variant, according to the number of different loci observed between the STs (one or two, respectively).

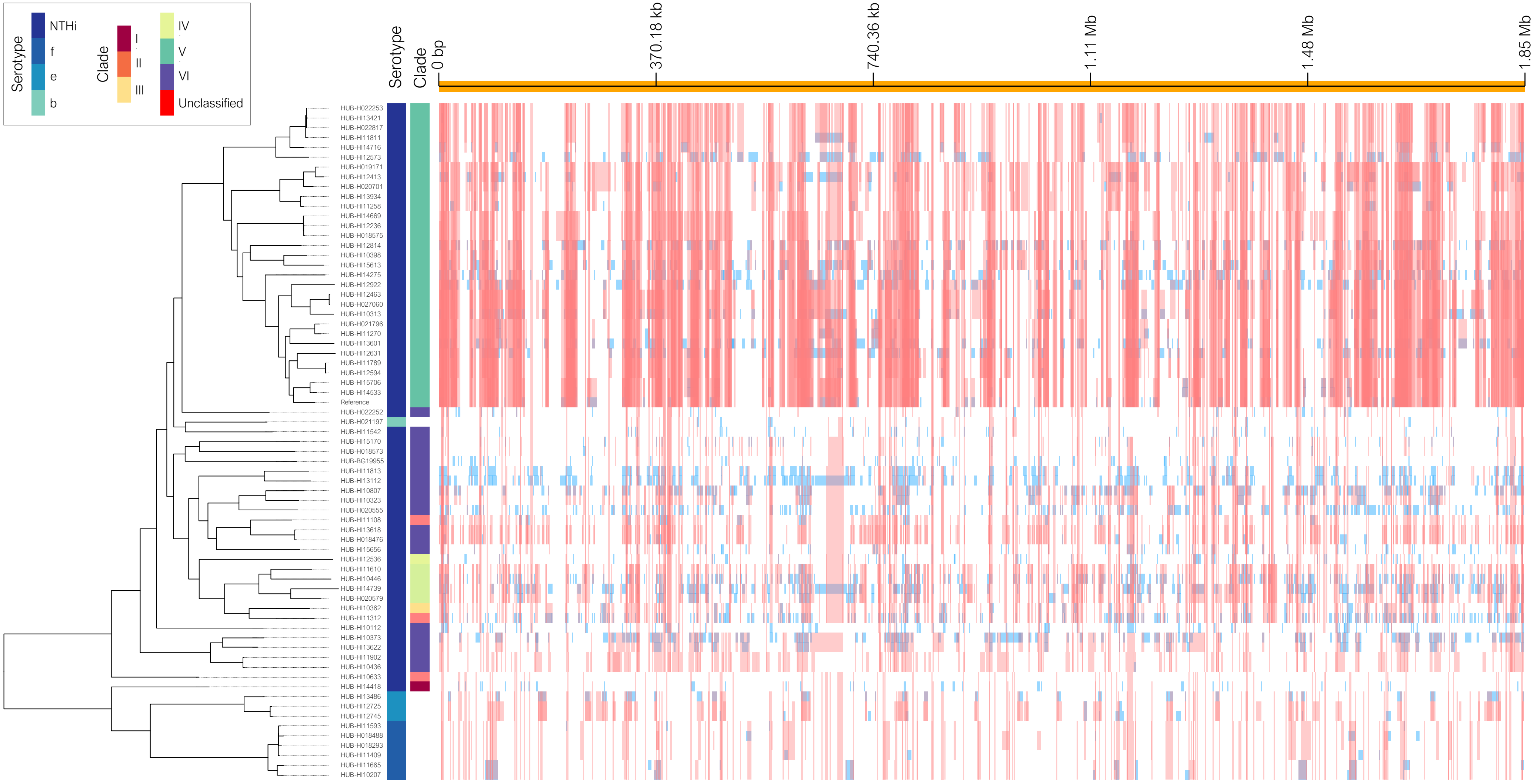

Figure S2. Sites affected by recombination in invasive *H. influenzae* isolates (2014-2019). Prediction of recombinant regions was performed using the Gubbins v2.3.1 software, and the core-SNP phylogenetic tree was built using RAxML-NG based only on shared positions after recombination removal. The Hi375 strain (CP009610) was used as a reference.

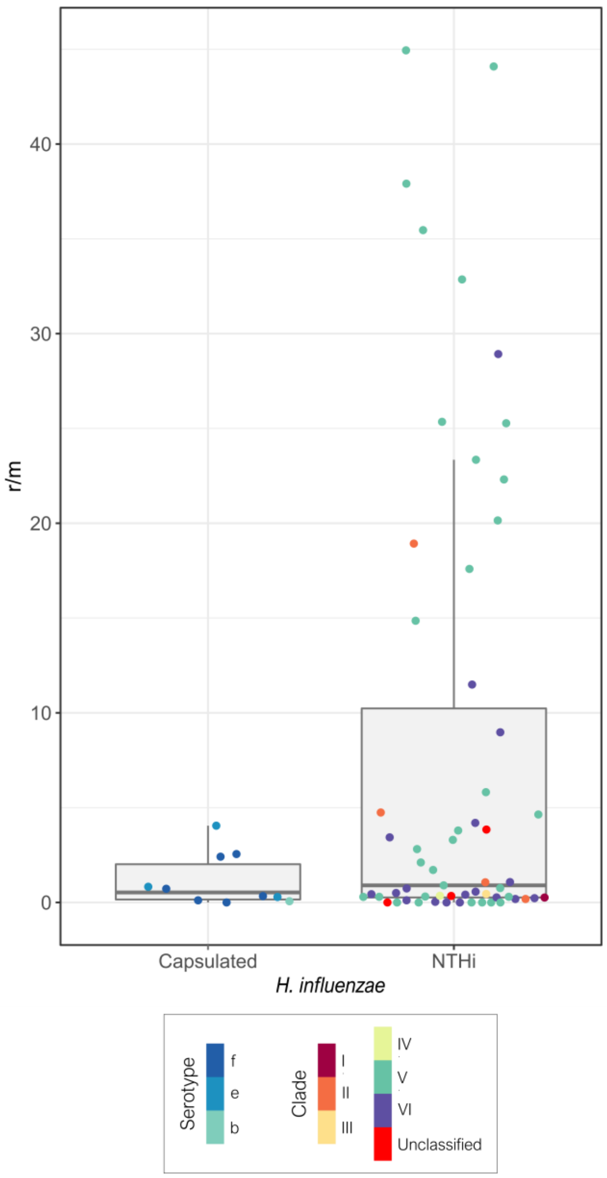

Figure S3. Relative impact of recombination and mutation (r/m) in invasive capsulated and NTHi strains (2014-2019). Prediction of recombinant regions was performed using the Gubbins v2.3.1 software, and the core-SNP phylogenetic tree was built using RAxML-NG based only on shared positions after recombination removal. The Hi375 strain (CP009610) was used as a reference.
